# Pattern Evolution during Double Liquid-Vapor Phase Transitions under Weightlessness

**DOI:** 10.3390/molecules22060947

**Published:** 2017-06-09

**Authors:** Ana Oprisan, Yves Garrabos, Carole Lecoutre, Daniel Beysens

**Affiliations:** 1 Department of Physics and Astronomy, College of Charleston, Charleston, SC 29424, USA; 2 ESEME,Institut de Chimie de la Matiere Condensee de Bordeaux, CNRS, Univ. Bordeaux, ICMCB, UPR 9048, F-33600 Pessac, France; 3 Service des Basses Temperatures, CEA-Grenoble et Universite Joseph Fourier, 38054 Grenoble, France; yves.garrabos@icmcb.cnrs.fr (Y.G.); carole.lecoutre@icmcb.cnrs.fr (C.L.); 4 Physique et Mecanique des Milieux Heterogenes, UMR 7636 CNRS-ESPCI-Universite Pierre et Marie Curie-Universite Paris Diderot, 10 rue Vauquelin, 75005 Paris, France; daniel.beysens@espci.fr

**Keywords:** phase separation, microgravity, binary coalescence

## Abstract

Phase transition in fluids is ubiquitous in nature and has important applications in areas such as the food industry for volatile oils’ extraction or in nuclear plants for heat transfer. Fundamentals are hampered by gravity effects on Earth. We used direct imaging to record snapshots of phase separation that takes place in sulfur hexafluoride, SF_6_, under weightlessness conditions on the International Space Station (ISS). The system was already at liquid-vapor equilibrium slightly below the critical temperature and further cooled down by a 0.2-mK temperature quench that produced a new phase separation. Both full view and microscopic views of the direct observation cell were analyzed to determine the evolution of the radii distributions. We found that radii distributions could be well approximated by a lognormal function. The fraction of small radii droplets declined while the fraction of large radii droplets increased over time. Phase separation at the center of the sample cell was visualized using a 12× microscope objective, which corresponds to a depth of focus of about 5 μm. We found that the mean radii of liquid droplets exhibit a $t^{1/3}$ evolution, in agreement with growth driven by Brownian coalescence. It was also found that the mean radii of the vapor bubbles inside the liquid majority phase exhibit a $t^{1/2}$ evolution, which suggest a possible directional motion of vapor bubbles due to the influence of weak remaining gravitational field and/or a composition Marangoni force.

## 1. Introduction

Phase transition is ubiquitous in nature, exhibited by processes such as the water cycle. However, when dealing with vapor-liquid phase transition, Earth gravity hampers a detailed study. The raising of the vapor bubbles and the falling of the liquid droplets prompt the formation of a flat meniscus by the gravity-induced coalescence of bubbles or droplets [1,2]. Experiments under the space conditions of weightlessness thus allow hidden mechanisms to be clearly evidenced. In addition, dealing with fluids near their critical point allows critical scaling universality to be used to generalize the results to all fluids. It also allows for critical slowing down to observe phenomena that are usually quite difficult to analyze because of their very fast dynamics. The DECLIC (Dispositif pour l’Etude de la Croissance et des Liquides Critiques) flight model facility is a multi-user facility to investigate critical fluids’ behavior and directional solidification of transparent alloys. This is a joint NASA and CNES research program onboard the International Space Station (ISS). The compact design contains three inserts, of which we refer here only to the ALI (Alice Like Insert) dedicated to the study of sulfur hexafluoride (SF_6_) as a near-ambient temperature critical fluid. The program covers a whole characterization of SF_6_, ranging from thermodynamic quantities measurements (thermal diffusivity, heat capacity and turbidity near the critical point) to boiling effects studies [3].

The focus of this paper is on investigating the phase separation process that takes place after the supercritical SF_6_ sample is brought from an initial state, which is already in a liquid-vapor two-phase state slightly below the critical temperature, to a temperature 0.2 mK below its initial temperature by a temperature quench [4,5]. Generally, phase separation could occur in a classical mean-field approach via two mechanisms: nucleation and spinodal decomposition, respectively [6,7,8,9]. It has been established that nucleation occurs in metastable systems [9,10,11,12], while spinodal decomposition occurs in thermodynamically unstable systems [13]. Dynamics at late time is governed either by Brownian-induced coalescences of bubbles or droplets or by long-ranged hydrodynamic interactions resulting in interconnected droplet or bubble patterns. The equilibrium volume fraction of the minority phase determines whether the system phase separates via disconnected or interconnected droplet/bubble pattern [8,14,15]. This distinction is different than spinodal decomposition or nucleation, based on thermodynamic arguments. Moreover, the presence of solid walls and the wetting effects dramatically modify phase separation dynamics [16,17,18,19,20,21]. Both phase separation and critical wetting phenomena were studied almost independently, and it has been shown that these two non-equilibrium phenomena could be coupled [22,23]. Wetting dynamics is coupled with phase separation that takes place in the bulk of the fluid. In the case of liquid droplet-forming phase separation, the rate of the growth of the wetting layer is much slower than in the case of bicontinuous phase separation [16,24].

**Figure 1:**
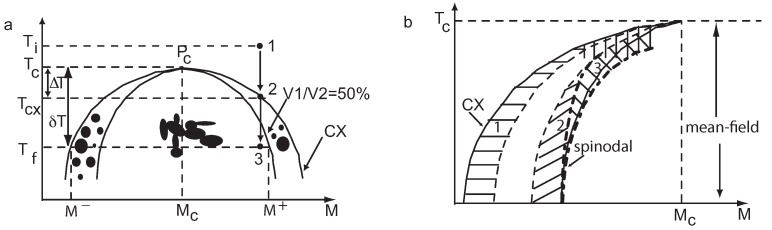
(**a**) Classical picture of growth and morphology phase diagram of SF_6_. The supercritical fluid at initial temperature $T_{i}$ (Point 1), with an off-critical density $\delta M=M-M_{c}$ is quenched below the coexistence (CX) curve, and droplets of vapor (volume V1) and liquid (volume V2) nucleate and grow. The curve $V_{1}/V_{2}=1/2$ separates the region of slow growth driven by Brownian collisions (1→2, quench 50 mK below CX) with disconnected droplets from the fast growth region limited by a chain reaction of coalescence (1→3, quench 3 mK below CX) with interconnected droplets. The critical point $P_{c}$ is characterized by critical temperature $T_{c}=45.557297$ ${}^{\circ }$C, and the order parameter $M_{c}$ corresponding to the critical density $\rho_{c}$ = 0.737 g/cm${}^{3}$; (**b**) The phase diagram of the generalized nucleation and growth. The regime between the coexistence curve and the left of the two broken lines (marked 1) is described by the classical nucleation theory. In this regime, a further (smooth) crossover from mean-field-like critical behavior to non-mean-field behavior occurs. The regime between the right broken curve and the left dashed-dotted curve (marked 2) is the regime of spinodal nucleation, which only exists in the regime of mean-field critical behavior. The regime inside the two dashed-dotted lines (marked 3) around the spinodal curve is the regime where a gradual transition from nucleation to spinodal decomposition occurs. Closer to $T_{c}$, the effect of large density fluctuations leads to a regime of non-metastable, generalized nucleation.

When quenched from the initial temperature $T_{i}>T_{c}$ to a final temperature $T_{f}<T_{c}$, the lever rule relates the volume fraction of the minority phase, ϕ, of the vapor and the order parameter *M* [14,15,25,26]:

(1) $$\phi =\frac{M^{+}-M}{M^{+}-M^{-}}=\frac{1}{2}\left(1-{\left(1+\frac{\Delta T}{\delta T}\right)}^{-\beta }\right)$$

where $\delta T=T_{c}-T_{f}$ is the quench depth with respect to the critical temperature, *T_c_* = 45.557297 °C, $\Delta T=T_{c}-T_{cx}$ is the coexistence temperature depth with *T_cx_* = 45.557270 °C, *β* = 0.32575 is a universal exponent and $M^{\pm }$ are the order parameters (relative densities) of liquid and vapor phase, respectively. This is defined as $M^{\pm }=\left(\rho^{\pm }-\rho_{c}\right)/\rho_{c}$, where $\rho^{\pm }$ is the mean density of liquid and vapor phase, respectively, and $\rho_{c}$ is the critical density [25,27]. The coexistence curve (CX) (see Figure 1a) is given by $M=B{\left(1-T/T_{c}\right)}^{\beta }$, where B=1.596 [28].

In his seminal work on nucleation and growth processes, Gibbs [29,30] considered that small nuclei occur randomly with state parameters similar to the properties of the newly-evolving macroscopic phases. In the classical scenario of nucleation and growth, only large fluctuations can overcome the energy cost of the formation of the interface of a nucleus of the new phase (the region between the spinodal and the coexistence curves in Figure 1a).

In van der Waals’ [31] and Cahn-Hilliard’s [32,33,34] visions of the spinodal decomposition, a set of long-wavelength fluctuations of the density of the initial state undergo spontaneous growth over large regions in space. Spinodal decomposition occurs when a fluid is quenched into the center of its coexistence region and separates into vapor and liquid phases (the region under the spinodal curves in Figure 1b). Immediately after the quench, density fluctuations whose length scale is approximately the correlation length ξ become unstable and increase in amplitude through diffusion. During this early stage of phase separation, the domain size increases as $t^{1/3}$[34,35,36]. As the phases reach nearly equilibrium density, the interfaces between the phases sharpen to a thickness of order ξ. Ultimately, minimization of interfacial area drives viscous flows resulting in a coarsening growth of $t^{1}$ [34,35]. This behavior has been observed in molecular fluids [37], binary fluid mixtures [38] and polymer blends [39].

Although both Gibbs’ and van der Waals’ description of heterogeneous systems are considered essentially correct and equivalent, the growth laws of the critical clusters in metastable systems obtained with the two theories are in deep contradiction [9,11]. Recently, it was suggested that large fluctuations of the order parameter, i.e., density in the pure fluids, induce generalized nucleation processes, which is intermediate between the spinodal decomposition and the nucleation (see Figure 1b) [10,11,12,40]. Late stages of growth, when phase separating domains are at near equilibrium, is not dominated any more by diffusion but by hydrodynamics. For volume fraction larger than typically $V_{1}/V_{2}=1/2$, hydrodynamic flows during a coalescence process between domains is able to induce another coalescence events, and so on, resulting in a coalescence chain reaction and phase transition dynamics only limited by the coalescence flow; domain typical size thus grow as t1 and the phase separating pattern is interconnected. For volume fractions $V_{1}/V_{2}<1/2$, the range of hydrodynamic flows is not large enough to induce domain collisions, which are now governed only by the haphazard of Brownian motion. Liquid droplets and vapor bubbles, respectively, grow slower in $t^{1/3}$ and the pattern is disconnected as an assembly of drops or bubbles [41]. Note that, since spinodal decomposition occurs in the center of the coexistence curve and nucleation near the coexistence curve branches, these two phase transition dynamics, fast and interconnected, respectively, slow and disconnected, are often misleadingly confused with spinodal decomposition and nucleation processes.

In this paper, we report the first experimental observation of a double phase separation process in a pure fluid under microgravity conditions. The temperature quench before this 0.2 mK already brought the system barely below its critical temperature and produced an initial phase separation between the majority liquid phase and the vapor phase. The 0.2-mK temperature quench produced further phase separation in both liquid and vapor domains where we observed: (1) vapor bubbles inside the majority liquid phase, which wets the walls of the sample cell unit; and (2) liquid droplets inside the initially separated vapor phase. We analyzed this double phase separation process and obtained quantitative evolutions for the phase-separated liquid droplets and vapor bubbles, respectively, based on their statistics.

## 2. Wide Field of View Images Results

**Figure 2:**
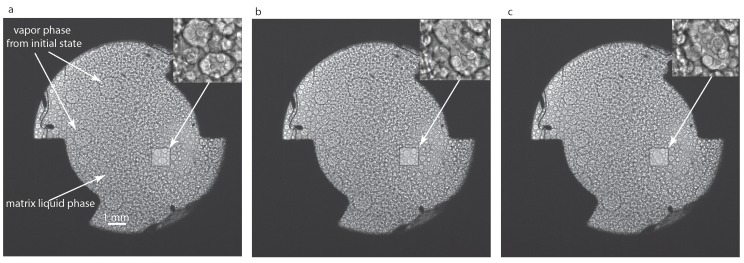
Binary coalescence in wide field of view (WFOV) images at successive frames: 6858.78 s (**a**), 6949.48 s (**b**) and 7040.35 s (**c**). The highlighted squares show the region of interest that contains two large liquid droplets of quite similar radii that approached each other(**a**) and then form a continuous droplet (**b**), which slowly changed to an ellipsoid (**c**) and finally becomes spherical (not shown). The insets of 1 mm × 1 mm size show a magnified version of the region of interest to better visualize the binary coalescence. The initial state was slightly below $T_{c}$ and produced an initial phase separation into a matrix liquid phase and some still visible vapor phase from the initial state.

We used iSolution^TM^ software to identify the droplets in the recorded images and Origin Lab^TM^ software for histogram extraction and data analysis. We only observed binary coalescence between liquid droplets in all wide field of view (WFOV) images that, over time, led to a significant change in the distribution of droplet radii (see Figure 2). Initially, the system was slightly below $T_{c}$ and phase-separated into a matrix liquid phase and some still visible vapor phase from the initial state. By further cooling down the system with the 0.2-mK temperature quench, a double phase separation process occurs in which we observe liquid droplets inside the initial islands of the vapor phase and also noticed the existence of vapor bubbles inside the matrix liquid phase. The coalescence shown in Figure 2 takes place inside of an initially phase-separated large and irregularly-shaped vapor bubble. The highlighted structures in the insets of Figure 2 are small liquid droplets that collide and form a larger liquid droplet.

**Figure 3:**
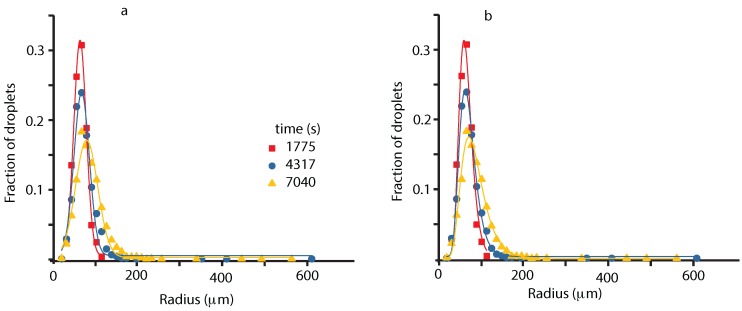
Histograms of WFOV images at successive times: 1775 s (solid squares), 4317 s (solid circles) and 7040 s (solid triangles). The continuous lines show the corresponding Gauss (**a**) and lognormal (**b**) fits. For the image at 1775 s (solid squares), the center of Gauss distribution is at $x_{cG}=\left(50.6\pm 0.5\right)$ μm and of the lognormal is at $x_{cLN}=(63.8\pm 1.2$) μm. The corresponding standard deviations were $w_{G}\;=\;(14.9\pm 0.7$) μm and $w_{LN}=(5.0\pm 0.4$) μm. For the image at 4317 s (solid circles), $x_{cG}\;=\;(66.3\pm 0.2$) μm and $x_{cLN}=(68.1\pm 4.8$) μm with the standard deviations $w_{G}=(18.4\pm 1.1$) μm and, respectively, $w_{LN}=(3.5\pm 0.1$) μm. For the image at 7040 s (solid triangles), $x_{cG}=(78.3\pm 1.2$) μm and $x_{cLN}=(81.3\pm 0.8$) μm with $w_{G}=(26.5\pm 1.2$) μm and $w_{LN}=(4.2\pm 0.1$) μm.

We selected a few representative WFOV images and check the fit of their corresponding histograms either with a Gaussian (see Figure 3a) or a lognormal (see Figure 3b).

There is a small increase in the coefficient of determination, which is a statistical measure of the goodness of the fitting process, which slightly favors the lognormal fit (see Figure 3). Another reason to favor a lognormal distribution is the presence of the long, asymmetric tail at large radii.

**Figure 4:**
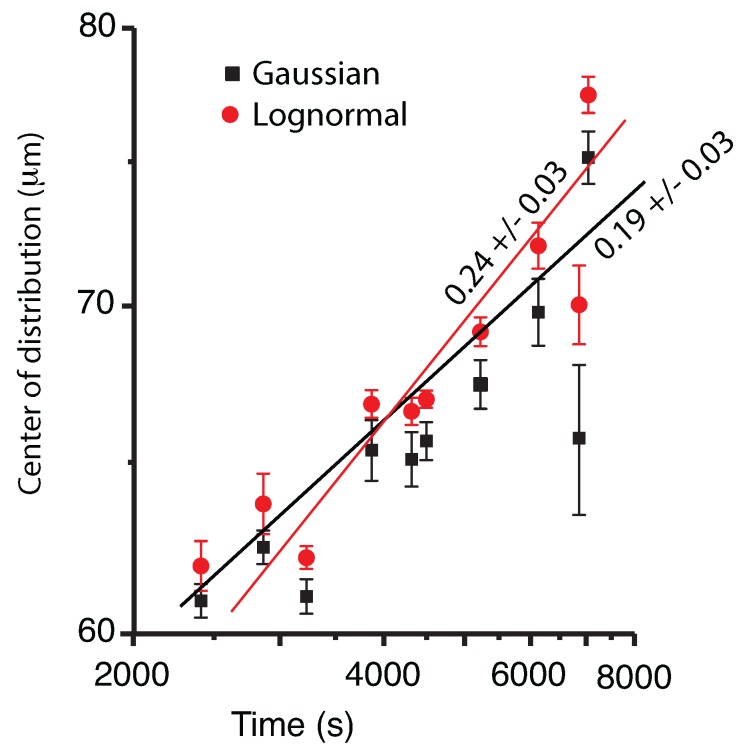
The location of the center of the Gaussian (solid squares), respectively, lognormal (solid circles) distribution versus time for WFOV images. The center of the distributions constantly shifts towards larger radii. The log-log plot suggests possible power laws for radii evolution.

We noticed that both the Gaussian and the lognormal fit show similar trends, i.e., the center $x_{c}$ of the distribution drifts towards larger radii over time. We hypothesized that the reason for such a drift is the observed coalescence that merges droplets into a larger droplet (see Figure 4). The fit of the log-log plot shown in Figure 4 suggests that the mean radius increase follows a power law with the exponents 0.19 ± 0.03 for the Gaussian fit (solid squares in Figure 4) and with an exponent of 0.24 ± 0.03 for the lognormal fit (sold circles in Figure 4). As Figure 4 shows, the error bars for the Gaussian fit are generally larger than the corresponding error bars for the lognormal fit. This is because the symmetric Gaussian curve cannot accurately capture the long tail of the radii distribution, especially during the later stages of the phase separation (see the large error bar of the Gaussian fit outlier at about 7000 s).

**Figure 5:**
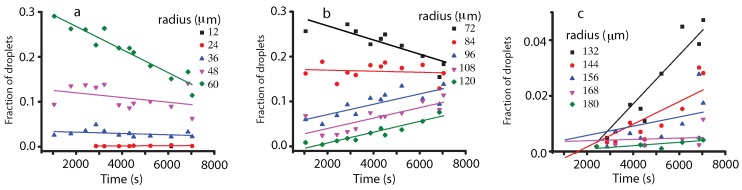
The temporal evolution of different bins of 12 μm (one pixel) in WFOV (solid square), 24 μm (solid circle), 36 μm (solid triangle), 48 μm (solid inverted triangle) and, respectively, 60 μm (solid inverted triangles) pixels (**a**); 72 μm (solid square), 84 μm (solid circle), 96 μm (solid triangle), 108 μm (solid inverted triangle) and, respectively, 120 μm (solid inverted triangles) pixels (**b**); and 132 μm (solid square), 144 μm (solid circle), 156 μm (solid triangle), 168 μm (solid inverted triangle) and 190 μm (solid inverted triangles), respectively (**c**). The smooth interpolation lines suggest some trends in different ranges of droplet radii.

In order to get a deeper insight into the droplet coalescence, we followed the temporal evolution of different one-pixel (12 μm) bins of the histogram (see Figure 5). While the 12-μm (1 pixel) and 24-μm (2 pixels) bins have very low contributions to the histograms, their relative fractions remained almost constant throughout the experiment. This suggests that either droplets of such radii form by coalescence from droplets of smaller radii that are not detectable (below one-pixel resolution) or they directly and constantly nucleate from the vapor phase. The 36-μm (3 pixels), 48-μm (4 pixels), 60-μm (5 pixels) and 72-μm (6 pixels) droplets show a clearly distinct pattern with a descending trend that suggests a steady depletion of these small radii droplets, due to coalescence that leads to larger droplet formation. The 84-μm (seven pixels) radii droplets seem to remain almost constant over time and constitute some kind of a borderline between the constantly decaying fraction of smaller droplets and the constantly increasing fraction of larger than 84 μm (seven pixels) droplets. All droplets with the radius over 96 μm (8 pixels) have a positive trend, which means they are generated from smaller droplets (see Figure 5b). Although the contribution of droplets larger than 132 μm (11 pixels) is relatively small (see Figure 5c), it is worth mentioning that they seem to appear predominately towards later stages of the phase separation. This is consistent with the concurrent and sustained increase of droplets of a large size 96–120 μm (8–10 pixels) observed in Figure 5b. It is only after enough such 96–120-μm (8–10 pixels) droplets formed that the large droplets (≥11 pixels) start to show up on our macroscopic images. Such a behavior suggests a cascade, or recursive, process during which binary coalescence between small droplets slowly generates a population of larger and larger droplets.

Although the fitting evolution of different radii bins in Figure 5 was solely provided as a visual aid to help the reader eyeball the general trends, we also provide the fitting parameters below. The least square method for the linear fitting equation y=a+bx was used, and the adjusted R^2^ coefficient was provided as a measure of the goodness of fit (see Table 1).

## 3. Narrow Field of View Image Results

**Figure 6:**
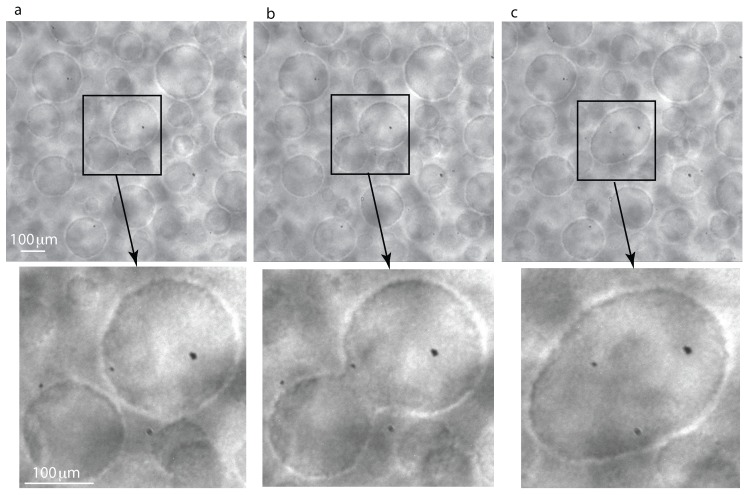
Binary collisions in narrow field of view (NFOV) images at successive frames: 6244 s (**a**), 6335 s (**b**) and 6426 s (**c**). The highlighted squares show two vapor bubbles of slightly different radii that approached each other (**a**) and then form a continuous bubble (**b**), which slowly changes it shape until it becomes spherical (**c**). We also magnified the region of interest for better visualization of coalescence.

A microscope with a magnification of 12× and a corresponding depth of focus of about 5 μm [42] that switches periodically between the WFOV and a small magnified portion of the direct observation cell (DOC) was also used. In the narrow field of view (NFOV), one pixel is equivalent to 0.977 μm. The fixed microscope objective focused at the center of the DOC happened to record two types of NFOV images. Initially, the microscope captured images from inside a large vapor bubble that phase-separated before we applied the 0.2-mK temperature quench (as seen in Figure 2 and labeled “vapor phase from initial state”, i.e., prior to applying the 0.2-mK temperature quench). Inside this large vapor bubble, coalescence between liquid droplets takes place. While the vapor phase recedes and the large vapor bubble seen on the right side of Figure 2 becomes smaller and smaller, the microscope captures images from the region marked “matrix liquid phase” in Figure 2. In this region, we also noticed coalescence as seen in Figure 6, but this takes place between the vapor bubbles embedded in the fluid majority phase. The high resolution recording and the slowing down of coalescence processes due to microgravity conditions allowed us to observe binary collisions as in Figure 6.

**Figure 7:**
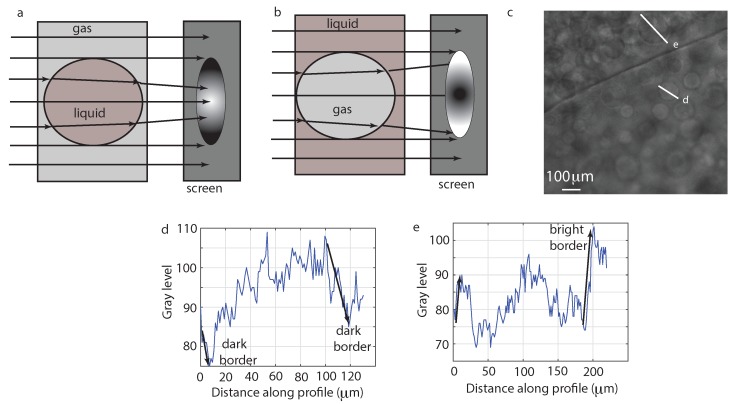
(**a**) Liquid droplets embedded in a vapor bubble tend to focus the parallel rays towards their center when projected on a screen (CCD camera); (**b**) vapor bubbles inside a liquid phase defocus the parallel beam and tend to produce a darker region towards the center of the bubble; (**c**) the interface separates a large vapor bubble (lower portion of the image) from the liquid phase (upper portion); (**d**) the intensity profile line across a liquid droplet marked “d” in Panel c shows a sharp decline in brightness with a minimum at about 10 μm as the line crosses into the droplet and another sharp decline in brightness with another minimum at about 120 μm; (**e**) the intensity profile line across a vapor bubble marked “e” in Panel c shows a sharp increase in brightness with a maximum at about 10 μm as the line crosses into the vapor bubble and another sharp increase in brightness with another maximum at about 200 μm.

We closely inspected the structures formed both inside the large vapor bubbles and inside the majority liquid phase and noticed some consistent patterns. Inside the vapor bubbles, the structures that coalesce have a dark contour and brighter interior, which suggests that they are liquid droplets. The structures inside the majority liquid phase (such as those shown in Figure 6) have a bright contour and a darker interior, which suggest that they are vapor bubbles. According to our sketch of the ray tracing shown in Figure 7a, parallel rays passing through a large vapor phase with liquid droplets inside it would be focused by the liquid droplets. As a result, the recorded image of the embedded liquid droplets has a dark boundary and is brighter inside. Similarly, parallel rays passing through a majority liquid phase with vapor bubbles inside it produces a divergent beam as in Figure 7b. In this case, the recorded image of the embedded vapor bubble should have a bright rim and darker towards its center. In Figure 7c, we show an image with a clear interface between the receding vapor bubble (lower portion of the image) and the expanding liquid phase (upper portion of the image). We used MATLAB’s pixel line profiler to show how the intensity changes across a large vapor bubble inside this liquid phase (see panel e). When the intensity profile line enters the vapor bubble from the upper left corner and moves downwards towards the lower right corner, the brightness increases (over about 10 pixels, which is about 9.77 μm), and then, when the line exits, the bubble there shows another sharp increase in brightness. The vapor bubble measures about 180 pixels (about 175.9 μm). The lower portion of the image in Figure 7c is the large vapor phase that constantly recedes (as seen in the WFOV images). In this region, the liquid droplets have a dark border and brighter interior consistent with the intensity profile shown in Figure 7d.

Although we might be tempted to consider that WFOV and NFOF should be similar, we need to keep in mind that the NFOV offers only a snapshot of a small portion of the system, and we can hardly make any assumption regarding the system’s homogeneity. Therefore, the distribution of droplets observed when counting all droplets in the WFOV images may not be identical to the one observed locally in the NFOV images. Even if the system were in thermal equilibrium, the spatial homogeneity hypothesis is hard to support as local displacements/perturbations could happen at any time, and they could rapidly and transiently change the observed droplets’ distribution. Furthermore, even if spatial homogeneity holds, there is the finite size effect of the microscopic window that limits the largest droplets that could be observed experimentally.

With all of these caveats in mind, we analyzed separately: (1) the distribution of the liquid droplets inside the large vapor bubble seen on the right side of Figure 2; and (2) the distribution of the vapor bubbles inside the majority liquid phase.

### 3.1. Liquid Droplets Dynamics from NFOV Images

Inside any large vapor bubbles, a dynamic process of continuous condensation of liquid droplets from the supercritical phase takes place. When we used the microscope to record NFOV images, only a small portion of the entire sample cell was visualized. As we noticed from the WFOV images, the large vapor bubbles are in continuous, slow motion due to internal processes, such as droplet nucleation and expansion of the wetting layer at cell boundaries, and also Brownian motion and g-jitter and remaining steady gravity due to the fact that the sample is not at the spacecraft center of mass. As a result of the macroscopic motion, some microscopic recordings caught the slow drift of the interface between a large vapor bubble (with liquid droplets inside) and the majority liquid phase (with vapor bubbles inside).

We fitted the liquid droplets’ NFOV distributions both to Gauss and lognormal functions to gauge possible trends over time (not shown). As we found out from analyzing WFOV images, there is no significant difference in the goodness of fit between the two fitting functions. However, we may favor the lognormal distribution due to the presence of the asymmetric, long tail in the experimental data.

**Figure 8:**
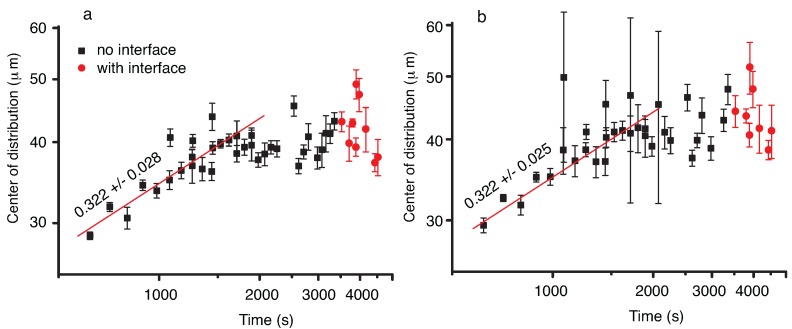
The location of the center of the Gaussian (**a**); and lognormal (**b**) droplet radii distributions shift initially towards larger values. At some point during the experiment, a large vapor bubble that contains the liquid droplets recedes such that the phase separation line drifted into focus (solid circles). While the best fit is still a lognormal distribution, at later times, the center of the distribution remains constant.

We also noticed that both the Gaussian and lognormal fit of the liquid droplets NFOV images show similar trends, i.e., the center of the distribution drifts at first towards larger radii (see Figure 8). Such a behavior of the NFOV droplets distributions correlates with the WFOV view results and is characteristic for the droplet distributions measured in the bulk of the vapor bubble that contains the liquid droplets.

The solid circles in Figure 8 cover a frame range where we observed a phase separation line slowly drifting from the top to the bottom of the NFOV image as the large vapor bubble recedes. In Figure 8, we reported only the measurements done inside the large vapor bubble, i.e., below the phase separation line when it becomes visible.

It is expected that the geometry of the vapor bubble and the proximity of the phase separation line changes the distribution of droplets compared to the bulk distribution (see the solid squares in Figure 8). For example, as the phase separation line recedes, the visible area of the bubble is smaller and smaller, which limits the droplet radii that could be observed.

The most noticeable result regarding droplet radii distribution is that initially the center of the distribution shifts towards larger and larger values. This is consistent with the coalescence mechanism and the radii increase as $t^{1/3}$ due to coalescences induced by Brownian collisions (see also [36]). After the initial fast increase in the average radii, the distributions flattened, which suggests that the process reached a steady state.

**Figure 9:**
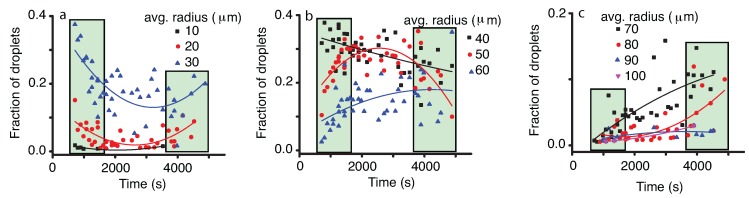
The evolution of different bins of 10 μm centered at the radii of 10 μm (solid square), 20 μm (solid circle), 30 μm (solid triangle) (**a**); 40 μm (solid square), 50 μm (solid circle), 60 μm (solid triangle) (**b**); and 70 μm (solid square), 80 μm (solid circle), 90 μm (solid triangle), 100 μm pixels (solid inverted triangle) (**c**). The smooth interpolation lines suggest some trends in different ranges of droplet radii. The initial shaded rectangle (time below 1800 s) corresponds to the initial $t^{1/3}$ power law (see Figure 8). The intermediate range corresponds to the flat droplet distribution observed in Figure 8. The shaded rectangles at the end of the data correspond to distributions measured when the phase separation line was in focus.

A more detailed picture of the coalescence mechanism emerges when we investigate the evolution of different radii bins. For this purpose, all distributions were binned with 10 pixels (≈10 μm). To separate images that were entirely inside the vapor bubble (time below 3500 s) from those that contained the phase separation line (after 3500 s), the latter were shaded in Figure 9. Furthermore, to allow more flexibility in eyeballing the data trends, we fitted with a quadratic instead of a straight line like in Figure 5. Presumably, this would be helpful in separating trends in the bulk compared to trends in the images containing the phase separation line.

When we focus on the initial stage of coalescence in the NFOV (for times below 1800 s that correspond to the $t^{1/3}$ trend shown in Figure 8), then from Figure 9a,b, it seems that for droplets of radii below 40 μm, there is a general descendent trend. At the same time, from Figure 9b,c, the radii larger than 50 μm seem to have an ascendant path (see only the first shaded rectangle at times below 1800 s). This evolution could be the result of coalescence events that deplete the distribution of small droplets in favor of generating larger droplets.

During the steady state plateau when the center of the droplet distribution remains almost fixed in Figure 8 (corresponding to the time interval between 1800 s and 3500 s), it seems that the distribution for droplets smaller than 40 μm continues to decline at a slower rate, whereas the intermediate range of 50 and 60 μm remains almost flat. Only the droplets larger than 70 μm seem to continue increasing their relative fraction.

Finally, during the images that contained the phase separation line, the small droplets fraction seems to increase again. However, this could be simply a measurement artifact due to the finite size effect of the observed area since larger droplets could not be accommodated and observed inside a continually decreasing piece of vapor bubble.

Again, the fitting evolution of different radii bins in Figure 9 was solely provided as a visual aid to help the reader eyeball the general trends. The Levenberg–Marquardt algorithm with the parabolic fitting equation $y=a+bx+cx^{2}$ was used, and the adjusted R^2^ coefficient was provided as a measure of the goodness of fit (see Table 2).

### 3.2. Vapor Bubbles Dynamics From NFOV Images

**Figure 10:**
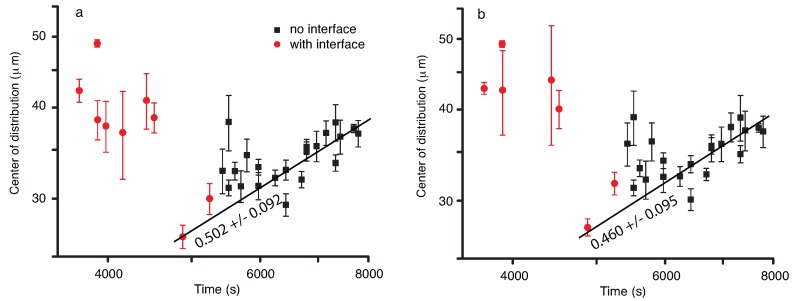
The location of the center of the Gaussian (**a**), respectively lognormal (**b**); vapor bubble radii distributions shifts towards larger values at times above 5000 s when the phase separation line is no longer in the field of view (solid squares). The radii seem to increase as $t^{1/2}$ in the bulk of the fluid phase. When the phase separation line is visible, the average radius seems to remain constant (solid circles).

Inside the liquid matrix, a dynamic process of phase separation of vapor bubbles from initial liquid phase takes place. As in the previous subsection, we also fitted the vapor bubbles’ NFOV distributions both to Gauss and lognormal functions to gauge possible trends over time (see Figure 10). Both the Gaussian and lognormal fit of the vapor bubbles’ NFOV images show similar trends. While the large vapor bubble (with liquid droplets inside) receded from the field of view, we recorded initially vapor bubble distributions in the presence of a moving phase separation line for times below approximately 5000 s. As we discussed in the previous subsection, such distributions (marked with solid circles in Figure 10) are skewed due to the finite size effects near the phase separation line. For this reason, we did not consider them when analyzing the trend of the mean radius versus time.

After the phase separation line is out of the field of view, we recorded images from the bulk of the liquid matrix that contained vapor bubbles undergoing coalescence. The most noticeable result regarding vapor bubbles radii distribution is that the center of the distribution shifts towards larger and larger values. This is consistent with the coalescence mechanism, and the radii increase as $t^{1/2}$.

**Figure 11:**
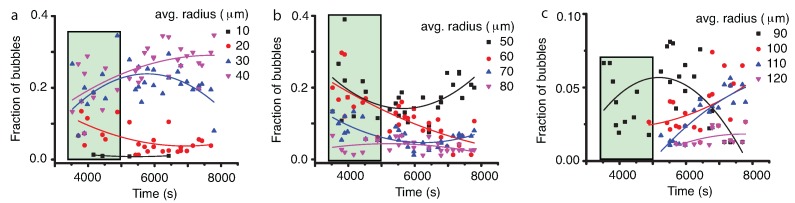
The temporal evolution of different bins of 10 μm centered at the radii of 10 μm (solid square), 20 μm (solid circle), 30 μm (solid triangle) and 40 μm (solid inverted triangle) (**a**); 50 μm (solid square), 60 μm (solid circle), 70 μm (solid triangle) and 80 μm (solid inverted triangle) (**b**); and 90 μm (solid square), 100 μm (solid circle), 110 μm (solid triangle), 120 μm pixels (solid inverted triangle) (**c**). The smooth interpolation lines suggest some trends in different ranges of vapor bubble radii (see Table 3). The initial shaded rectangle (time below 5000 s) corresponds to the images containing the phase separation line.

A more detailed picture of the coalescence mechanism emerges when we investigate the evolution of different radii bins. To separate images that contained the phase separation line (time below 5000 s) from those recorded in the bulk of the liquid phase (after 5000 s), the former were shaded in Figure 11. If we focus on the initial stage of coalescence in the NFOV (for times below 5000 s), it seems that the dynamics is similar to the one observed across the phase separation line inside the large vapor bubble (see Figure 11, the last shaded rectangles). During the ascending trend of the average radius (see Figure 10), the fraction of the small droplets below 30 μm seems to decay over time (see Figure 11a) and slightly increase for intermediate 40–50-μm bubbles. Larger bubbles with radii from 60 μm–90 μm seem to also decrease their relative fraction, whereas large droplets over 100 μm increase their relative contribution to the distributions (see Figure 11c). This evolution could be the result of coalescence events and has a different dynamics than previous measurements in liquid droplets (see the previous subsection).

Similarly, the fitting evolution of different radii bins in Figure 11 was solely provided as a visual aid to help the reader eyeball the general trends. The Levenberg–Marquardt algorithm with the parabolic fitting equation $y=a+bx+cx^{2}$ was used, and the adjusted R^2^ coefficient was provided as a measure of the goodness of fit (see Table 3).

The temporal evolutions of the transition rates for radii distribution is relevant because, in the future, we would like to derive a phenomenological approach to droplet distribution and their dependence on different thermophysical parameters. It is known that for large droplet diameters and binary coalescence, the rate of change of the number of domains was estimated as $dN/dt=-N^{2}\int_{\Sigma }p\left(r\right)V\cdot nd\Sigma$, where *N* is the number of domains per unit volume; p(r) is the pair distribution function of the tubes/droplets; V is the relative velocity of the tubes/droplets; and n is the outward normal to the collision surface Σ [24,43]. They found that the long tail of the droplet size distribution, such as those found in both our WFOV and NFOV images, can be reasonably modeled mathematically for radii over a natural cutoff with a power law, i.e., $dN\left(r\right)/dr=Ar^{\theta }$ [43,44]. Molecular dynamic simulations showed that, for large volume fractions of the minority phase, the distribution of droplets versus their corresponding diameters becomes wider over time (see Figure 10b in [44]).

Our study opens the possibility of modeling liquid droplet and vapor bubble dynamics, respectively, not only in the limit of large radii, but for all radii, and could offer a more complete quantitative view of coalescence processes during phase separation.

## 4. Discussion

Double phase separation was previously observed both experimentally [45,46] and numerically [47] in fluid mixtures under the influence of surface fields. It has been found that the interface quench could induce spontaneous double phase separation under a geometrical confinement. It must be stressed that the mechanism for double phase separation in binary mixtures is different from the observed double phase separation in pure fluid SF_6_ reported here. In binary mixtures, the double phase separation was determined by high fluidity, which leads to rapid geometrical coarsening of domains due to a hydrodynamic process [47]. Because the diffusion process is much slower than the hydrodynamic one, the secondary phase separation is induced by local nonequilibrium processes.

In this study, an initial temperature quench brings the system slightly below its critical temperature and separates it into vapor and liquid phases. The subsequent 0.2-mK temperature quench produces a secondary phase separation inside each of the already separated phases. The droplet radii evolution with $t^{1/3}$ (see Figure 8) could be explained based on coalescence-induced Brownian collisions [25,41] since the domains are at nearly equilibrium. The Binder and Stauffer (BS) diffusive mechanism [35,48,49] is thus not relevant here. According to Siggia [35], the droplet *n* density evolves as $dn/dt=-Bn^{2}$, where *B* is the product of the droplet radius *r* and the droplet diffusivity D(r). However, under the assumptions of the generalized Stokes–Einstein–Sutherland relation [50,51], *B* could be considered a constant; with $n\propto r^{-d}$, where *d* is the space dimension. By substituting the above relationship into Siggia’s rate equation, one finds that the solution is $r\propto t^{1/d}.$ For a 3D space (d=3), the above scaling law gives an exponent of 1/3, which is also compatible with the evaporation-condensation mechanism proposed by Lifshitz and Slyozov [52], but not relevant here as the domains are at near equilibrium. For a more specific discussion regarding the difference between the Lifshitz and Slyozov mechanism [52] and phase separation produced via nucleation of liquid droplets and collisions among them, see Roy and Das [53,54]. A similar $t^{1/3}$ power law was obtained using molecular dynamics simulations of a single component two-dimensional Lennard–Jones fluid [55].

While Brownian collisions among liquid droplets seems to be the mechanism for liquid droplets’ coalescence that explains the $t^{1/3}$ power law [23,45,47,56,57,58,59,60] observed here (see Figure 8), it results that a different mechanism must be responsible for the observed faster evolution of vapor bubble radii with $t^{1/2}$ (see Figure 10). Recent numerical simulations [60] showed clear evidence of directional (as opposed to random Brownian) motion of droplets towards a neighboring droplet during phase separation. It has been hypothesized that the faster than $t^{1/3}$ droplet radii growth laws predicted by the BS mechanism are due to a composition Marangoni force [36,60] . Note that g-jitter and weak gravity are still present (the sample is not at the spacecraft center of mass), then it is difficult to put in clear evidence the Marangoni force.

## 5. Materials and Methods

**Figure 12:**
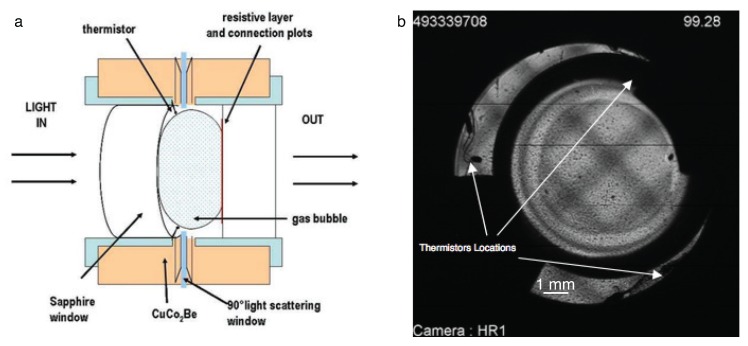
Schematic cross-section (not to scale) of the expected microgravity vapor-liquid distribution in the direct observation cell (DOC) dedicated to study the liquid film drying due to boiling phenomena, using a transparent resistive layer as a flat local heating source (**a**). Wide field and grid shadowgraph image of DOC at equilibrium in the two-phase range (**b**).

The DECLIC experimental setup was described extensively in previous publications [3,61]. Phase transitions near the critical point at room temperature, critical fluids and boiling crisis were studied in microgravity using the ALI insert. The optical pressurized cells (or the direct observation cell (DOC)) contained a heater device as a transparent resistive layer appropriate for light transmission observation. The DOC was filled with SF_6_ at its vapor-liquid critical point $T_{c}=318.737$ K, (45.587 °C), p=3.73 MPa and ρ=742.6 kg m^−3^. A sketch of the cell is shown in Figure 12a [62,63].

The fluid sample volume observed by light transmission corresponds to a cylindrical volume of D = 10.6 mm inner diameter and inner thickness *e* = 4.115 mm. The total fluid volume of the cell was 0.463 cm^3^ corresponding to a total SF_6_ mass of 0.353 g, leading to the filling mean density $\rho =\rho_{c}+2\%$.

Three small (250 μm bead diameter) thermistors (THERMOMETRICS B10, 10 kΩ resistance at 25 °C) are located inside the fluid volume, so that three local temperatures are measured close to the vapor-liquid interface in the microgravity environment and labeled R5, R6 and R7, respectively (Figure 12b).

The optical system allows interlaced recordings of both wide field of view (WFOV), which covers a circular area with diameter of 10.6 mm, and narrow field of view (NFOV), which only covers a 1×1 mm^2^ area located at the center of the DOC [61]. The light source was a 633-nm He-Ne laser.

**Figure 13:**
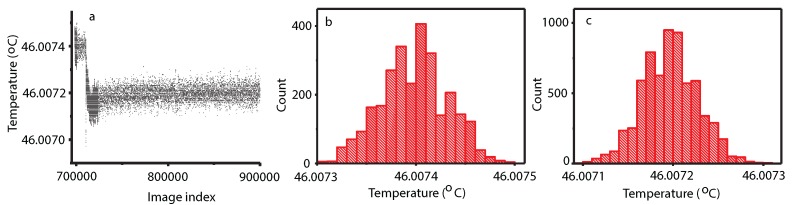
The DOC was slightly below the critical temperature when the temperature quench of 0.2 mK brought the system further below its critical temperature and produced a double phase separation (**a**); the thermistor readings are quite noisy, but the histogram of temperature readings for the images we analyzed is Gaussian with a mean initial value of 46.0074 ${}^{\circ }$C (**b**) and a mean of 46.0072 ${}^{\circ }$C after the 0.2-mK temperature quench (**c**).

The DOC received a temperature quench of 0.2 mK from its slightly subcritical coexistence temperature (see Figure 13a) that allowed the supercritical fluid to cross from the slightly phase-separated phase (see Figure 13c) into a double phase-separated system (see Figure 13d).

All data analyzed here belong to ALI Sequence 7 for which the temperature quench started at the image reference index of $t_{ref}$ = 1,123,710,576. The conversion for the clock is that the camera records Δ = 23 frames per second. For example, the image shown in Figure 2a has an image index of $t_{img}$ = 1,123,868,328, which means it was taken at $(t_{img}-t_{ref})/\Delta$ = (1,123,868,328−1,123,710,576)/23 = 6858.78 s from the temperature quench. The next image in Figure 2b was taken at 6949.57 s, and finally, Figure 2c was taken at 7040.35 s after the temperature quench.

## 6. Conclusions

We determined the temporal evolution of liquid droplets and vapor bubbles, respectively, distributions during a double phase separation process in pure SF_6_ as a result of a 0.2-mK temperature quench in microgravity. Both the WFOV and NFOV image analyses showed that the droplet distributions could be fitted with a lognormal function and that the peak of the distributions shifts towards larger droplet radii over time. The power law exponents for mean radii evolution as measured from WFVO images (see Figure 4) are about 0.2 (Gaussian fit) and, respectively, 0.24 (lognormal fit), which are slightly weaker than the expected 1/3 value for coalescence-induced Brownian collisions. In contrast, the NFOV power law exponents for the liquid droplet evolution (see Figure 8) are 0.322 ± 0.028 (Gaussian fit) and, respectively, 0.322 ± 0.025 (lognormal fit) is in agreement with the above coalescence mechanism. In NFOV, the power law exponents for the vapor bubble evolution (see Figure 10) are 0.502 ± 0.092 (Gaussian fit) and, respectively, 0.460 ± 0.095 (lognormal fit). This power law suggests a faster, directional motion of vapor bubbles due to a weak remaining gravitational field and a possible composition Marangoni force [60].

By following the temporal evolution of narrow bins of droplet radii (one-pixel increment in WFOV and 10-pixel increment in NFOV) it is noticed that some distributions reduce their contribution over time (for small radii), whereas the relative contribution of large radii bins increases over time.

## Figures and Tables

**Table 1:** The fitting parameters (*a*, *b*) and the corresponding goodness of fit statistics (R${}^{2}$) for the trends shown in Figure 5 with continuous lines.

Radius (μm)	a ($10^{-2}$)	b ($10^{-5}\;s^{-1}$)	R${}^{2}$
12	0.06	0.03	0.16
24	3.52	−0.15	0.04
36	13.0	−0.53	0.06
48	31.9	−2.58	0.90
60	32.3	−2.04	0.77
72	17.2	−0.12	0.08
84	4.78	1.15	0.59
96	1.65	1.16	0.89
108	−1.64	1.20	0.81
120	−1.80	0.88	0.58
132	−0.65	0.41	0.03
144	0.24	0.17	0.14
156	0.34	0.02	0.44
168	−0.04	0.06	0.40
180	−0.32	0.13	0.43

**Table 2:** The fitting parameters (*a*, *b*, *c*) and the corresponding goodness of fit statistics (R${}^{2}$) for the trends shown in Figure 9 with continuous lines.

Radius (μm)	a ($10^{-2}$)	b ($10^{-5}\;s^{-1}$)	c ($10^{-9}\;s^{-2}$)	R${}^{2}$
10	2.79	−2.32	5.58	0.39
20	14.9	−9.89	18.8	0.38
30	40.9	−17.4	27.0	0.41
40	35.3	−2.97	1.06	0.28
50	9.53	16.2	−31.6	0.43
60	4.64	6.3 0	−7.43	0.18
70	−1.76	3.99	−2.76	0.53
80	3.19	−2.26	6.8	0.43
90	0.10	1.10	−1.52	0.13
100	0.46	−0.06	1.181	0.13

**Table 3:** The fitting parameters (*a*, *b*, *c*) and the corresponding goodness of fit statistics (R${}^{2}$) for the trends shown in Figure 11 with continuous lines.

Radius (μm)	a ($10^{-2}$)	b ($10^{-5}\;s^{-1}$)	c ($10^{-9}\;s^{-2}$)	R${}^{2}$
10	8.78	−2.92	2.67	0.54
20	36.1	−9.32	6.72	0.27
30	−41.1	22.5	−19.5	0.21
40	−13.0	10.9	−7.05	0.36
50	75.0	−21.5	19.1	0.16
60	46.1	−8.40	3.94	0.59
70	41.7	−11.8	9.48	0.45
80	−5.11	3.62	−3.46	0.07
90	−16.0	8.38	−8.08	0.28
100	5.46	−1.58	1.98	0.15
110	−19.6	5.30	−2.76	0.72
120	−10.2	3.12	−2.03	0.06
